# High Prevalence of Multidrug Resistant Bacteria in Cirrhotic Patients with Spontaneous Bacterial Peritonitis: Is It Time to Change the Standard Antimicrobial Approach?

**DOI:** 10.1155/2019/6963910

**Published:** 2019-05-13

**Authors:** Jerônimo De Conto Oliveira, Enrique Carrera, Roberta C. Petry, Caroline Deutschendorf, Augusto Mantovani, Samantha Thifani Alrutz Barcelos, Santiago Cassales, Fernando Comunello Schacher, Antônio Barros Lopes, Mario R. Alvares-da-Silva

**Affiliations:** ^1^Post-Graduate Program in Gastroenterology and Hepatology, Universidade Federal do Rio Grande do Sul, Porto Alegre, Brazil; ^2^World Gastroenterology Organization Porto Alegre Training Center, Porto Alegre, Brazil; ^3^Infectious Disease Control Commission, Hospital de Clínicas de Porto Alegre, Porto Alegre, Brazil; ^4^Division of Gastroenterology and Hepatology, Hospital de Clínicas de Porto Alegre, Porto Alegre, Brazil

## Abstract

**Introduction:**

Spontaneous bacterial peritonitis (SBP) has a deleterious clinical impact in end-stage liver disease, and multidrug resistance has increased, raising concern about effectiveness of traditional antibiotic regimens.

**Patients and Methods:**

Single-center retrospective study of ascitic fluid infections in cirrhotic patients.

**Results:**

We analyzed medical records related to 2129 culture-positive ascitic fluid and found 183 samples from cirrhotic patients. There were 113 monobacterial SBP cases from 97 cirrhotic patients; 57% of patients were male; hepatitis C and alcohol were the main etiologies for cirrhosis. Multidrug resistant bacteria were isolated in 46.9% of SBP samples, and third-generation cephalosporin and quinolone resistant reached 38.9% and 25.7% of SBP cases.

**Conclusion:**

SBP due to multidrug resistant bacteria is a growing problem, and one should consider reported resistance profiles for the decision-making process of empirical first-line treatment prescription.

## 1. Introduction

Bacterial infection is one of the most important causes of acute decompensation and death in cirrhosis [1]. Recently, a growing body of research has described an increase in bacterial resistance in both general (e.g., pneumoniae) and specific (i.e., spontaneous bacterial peritonitis: SBP) infections in cirrhotic patients [2–8]. A number of traditional (e.g., previous hospitalization, nosocomial infection) and specific risk factors (e.g., quinolone use for SBP prophylaxis) have been related to drug-resistant bacterial infections in cirrhosis [2, 3]. Besides resistance, a shift to gram-positive germs have also been shown, especially in SBP, which has been related to invasive procedures during hospitalization [7–9].

Ascitic fluid infection is a deleterious event in cirrhotic patients [9–11]. Its changing pattern of germs puts clinicians and infectious disease control centers in alert about the appropriateness of traditional empirical antibiotics regimens, at least in some specific clinical settings. Even though ascitic fluid culture is frequently negative in patients suspected to have SBP [10], it is a powerful data to guide treatment when positive.

In this article, we describe the clinical and microbiological characteristics ascitic fluid infections in cirrhotic patients from a university hospital in Brazil. We aimed to estimate the prevalence of bacterial resistance against the main antibiotics and to identify risk factors for and the clinical impact of multidrug resistant pathogens infections. Moreover, we wonder to help to assess patterns in which traditional empirical antibiotic treatment may be ineffective.

## 2. Methods

This is a retrospective study performed at Hospital de Clínicas de Porto Alegre, a public tertiary academic hospital in the very south of Brazil. We retrieved microbiological and patient-related data from all positive ascitic fluid culture collected in the period between January 2010 and September 2017. We analyzed patients' electronic medical records and excluded pediatric and noncirrhotic patients and those with an episode of secondary peritonitis (defined by clinical data or polymicrobial cultures) [12].

Diagnosis of cirrhosis was defined by clinical, ultrasound, laboratory, and/or histological findings. Ascitic fluid infections were categorized according to the traditional criteria [10, 12, 13]: culture positive samples with a polymorphonuclear (PMN) cell count ≥250/mm^3^ were diagnosed as SBP, while bacterascites was defined with counts <250/mm^3^. We utilized The United States Centers for Disease Control and Prevention (CDC) and the European Centre for Disease Prevention and Control (ECDC) joint initiative terminology for antimicrobial-resistant bacteria [14]: multidrug resistance (MDR) was defined as acquired nonsusceptibility to at least one agent in three or more antimicrobial categories, while extensively drug-resistant (XDR) was defined as nonsusceptibility to at least one agent in all but two or fewer antimicrobial categories (i.e., bacterial isolates remain susceptible to only one or two categories).

We assessed serial variables in the study population: cirrhosis etiology, stage, and main clinical complications; previous admission, previous antibiotic treatment or prophylaxis against SBP, and time since hospital admission and paracentesis; terlipressin use for hepatorenal syndrome (HRS), hemodialysis, intensive care unit (ICU) admission, and death during hospitalization. Baseline clinical and laboratorial data were collected from the time of admission (preferably) or close to paracentesis date. Infections were considered to be nosocomial when diagnostic paracentesis was performed after more than 48h of hospital admission [12]. Missing data were excluded from statistical analyses.

Chi-square tests were used to compare proportions between groups. A P value of <0.05 was considered statistically significant. Statistical analyses were performed in SPSS (version 23.0; IBM Corp., North Castle, New York, USA). The report of this study is in consonance with the Strengthening the Reporting of Observational Studies in Epidemiology statement [15].

## 3. Results

**Figure 1:**
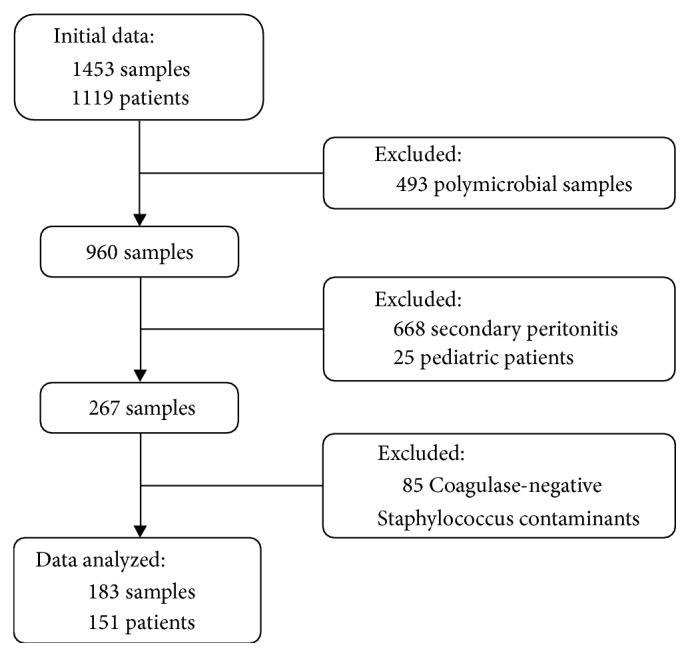
Flowchart of samples and patients.

In the hospital's electronic health records, we retrieved information from all 2129 positive ascitic fluid cultures collected from 1119 patients in the period of interest. After excluding polymicrobial cultures (related to secondary peritonitis), 960 monomicrobial samples from 784 patients remained. We assessed medical records from these patients and excluded 693 samples (668 related to secondary peritonitis and 25 from pediatric patients). Medical records from 206 cirrhotic patients (267 samples) were selected and finally deeply analyzed. Another 85 Coagulase-negative Staphylococcus not related to a SBP diagnosis were considered contaminants and were also excluded. After this process, 113 SBPs and 70 bacterascites episodes from a total of 151 patients were included in the study. A flowchart summarizes this information (Figure 1).

The demographic and clinical characteristics of our population are summarized in Table 1. Population were predominantly male (57%), with a mean age of 57 years (±12.9). Hepatitis C and alcohol (each isolated or in association) were the cause of cirrhosis in 84% of cases. Almost all patients had a Child-Pugh B (36.4%) or C (62.1%) score at the time of paracentesis, with a mean MELD score of 21.2 (±9.1), while 14.2% were on antibiotic prophylaxis against SBP. 82% of paracentesis were performed in already hospitalized patients, half of them in less than 72h from admission. A diagnosis of SBP was made 113 times (61.7% of samples) in 92 patients, and 48.7% of these were of nosocomial origin.

Gram-positive bacteria were isolated in 38.9% of SBP cases. MDR and XDR germs represented 46.9% of all SBP-related cultures (39.8% and 7.1%, respectively). MDR and XDR together were responsible for 39.6% and 53,7% of community acquired (CA) and nosocomial SBPs, respectively. We found no statistically significant difference in prevalence of resistant germs in patients with previous admission or antibiotic usage in the previous 30 days or on prophylaxis against SBP.

Ceftriaxone resistance was observed in 14% of CA SBP and 45% of nosocomial SBP cases. There was no quinolone resistance (reported together for levofloxacin and ciprofloxacin) in CA SBP, although it reached 49% among nosocomial SBP episodes. Specific resistances were analyzed and results for the most frequent germs are summarized in Tables 2 and 3.

Among patients with SBP, we found no statistically significant difference between those infected with a resistant bacterium compared to a nonresistant one in the following outcomes: ICU admission (70,7% x 59,2%), renal replacement therapy initiation (41,5% x 42,9%), and hospital mortality (57,3% x 58,1%).

## 4. Discussion

Bacterial resistance is a well-known problem with worldwide impact. Improvement in hospital care resulted in longer survival rates, but also had created a scenario with even more severe end-stage liver disease patients, in which there is more frequent and disabling infections. This have raised concern of MDR bacteria in cirrhotic patients [2, 3], which might be related to poorer prognosis. In this study, we found a high prevalence of multidrug and extensively drug-resistant bacteria either in community or nosocomial SBP.

Salerno et al. conducted a multicenter prospective study on multidrug resistant infections (not restricted to SBP) and reported a greater mortality of when compared to patients with antibiotic-susceptible infections [6]. Nonetheless, antibiotic resistance did not worsen SBP prognosis in other studies [4, 7]. In our population, we found no statistically significant difference in clinically relevant outcomes related to bacterial resistant SBP. Although this negative finding is in consonance with other reports on SBP, a beta error should be considered.

We did not find any significant relation in bacterial resistance for the analyzed possible risk factors, except for nosocomial acquisition of infection. Notably, previous studies also failed to show a statistically significant impact of antibiotic prophylaxis with norfloxacin or severity of cirrhosis on MDR bacteria prevalence in SBP [3, 16].

There are some limitations in our study that should be noted: it is a single-center retrospective study, with a convenience sampled population and limited reliable information on some clinical data from patients before hospital admission. Moreover, our study describes a very severe cirrhotic population, with almost all patients being classified as Child-Pugh B or C, two-thirds of them being admitted in the ICU and more than a half dying in the hospital, which may limit external validity at some extent. SBP usually settles in a set of end-stage liver disease, and culture-positive SBP usually presents in patients with a more severe background and carries a poorer prognosis than culture-negative neutrocytic ascites [17], which may have resulted in a selection bias and explain the more severe stage of our patients if compared to other publications. On the other hand, we analyzed a similar or even bigger number of patients than previous reports with similar methodology focused in SBP [2, 7, 8, 18] and performed a comprehensive assessment of microbiological data on ascitic fluid infections.

In the past decade, prevalence of MDR bacterial infections has increased substantially, what is consistent among serial studies from different geographical regions [2–8, 19]. In SBP infections, for instance, Fernández et al. (Spain, 2012) [3], Oliveira et al. (Portugal, 2016) [4], Costabeber et al. (Brazil, 2016) [5], Oey et al. (Netherlands, 2017) [7], and Sofjan et al. (USA, 2018) [18] have reported MDR prevalence of 22, 20, 37, 32, and 30%, respectively. We have found a slightly higher proportion than previous studies (39.8%), but it should be noted that this rate is considerably different when MDR and XDR germs are put together (46.9%) and reaches 53.7% when only nosocomial infections are considered.

If on the one hand MDR and XDR prevalence is greater than other published studies of our knowledge, on the other hand we found a similar or even smaller resistance to quinolones. This may be related to quinolone restrictions by our hospital infection control commission and a small proportion of patients being on prophylactic antibiotics against SBP.

Third-generation cephalosporins resistance is of particular interest, once this class is recommended as first-line empirical treatment by international guidelines [11, 13]. We reported a 22% overall resistance rate among SBP cases (14% and 45% for CA and nosocomial infections, respectively), which is inside the previous reported rates that ranged widely from 15% to 45% [5, 7, 18]. A recent meta-analysis [20] evaluated third-generation cephalosporin resistance in 8 studies and concluded that this class should be used with caution, especially in centers where resistance patterns are not available.

In our study, resistance against piperacillin-tazobactam (recently included on first-line alternatives by the European Association for the Study of the Liver [11]) occurred in a small proportion of patients (2% for CA and 15% for nosocomial infections); this emerges as a reliable first-line regimen, but consideration of local resistance profiles is always warranted [20].

We also showed a difference between gram-positive and gram-negative prevalence in ascitic infections, which may be related to reporting issues, once we analyzed and showed separate result for patients with SBP and bacterascites. Even though there is a growing incidence of gram-positive infections in cirrhotic patients [7, 8], true SBP seems to still be related to gram-negative bacteria.

Piano et al. [19] have recently analyzed the big picture of multidrug resistance in cirrhosis in a big multicenter worldwide prospective study in hospitalized patients with 1302 infections and 354 SBP cases, bringing new scientific data from different countries together.

Scrutiny of germs ascitic fluid cultures may guide antibiotic prescription and possibly reduce the burden of these infections. A consistent body of evidence is available on multidrug and specific resistances in cirrhosis. In the near future, it may be necessary for international guidelines to change recommendations on first-line empirical antibiotic treatments in SBP. It remains essential to have knowledge of local resistance patterns in order to orientate empirical antibiotic regimens or, at least, alert clinicians about possible loss of efficacy in traditional antibiotic regimens in certain high-risk patients.

## Figures and Tables

**Table 1:** Demographic and clinical characteristics of patients.

	All patients	Without SBP	With SBP
	(n = 151)	(n = 54)	(n = 97)
*Age mean (SD)*	57,4 (12,9)	61,8 (10,1)	55,2 (13,6)
*Gender n (%)*			
Female	65 (43)	22 (41)	43 (43)
Male	86 (57)	32 (59)	54 (57)
*Cirrhosis etiology n (%)*			
HCV	72 (49,7)	20 (40,8)	52 (54,2)
HCV+Alcohol	23 (15,9)	11 (22,4)	12 (12,5)
Alcohol	27 (18,6)	10 (20,4)	17 (17,7)
HBV	2 (1,4)	1 (2)	1 (1)
NASH	7 (4,8)	3 (6,1)	4 (4,2)
Other	14 (9,7)	4 (8,1)	10 (10,3)
*Child-Pugh score (value) mean (SD)*	10,2 (2,0)	9,1 (1,6)	
*Child-Pugh score (categ.) n (%)*			
A	2 (1,4)	2 (4,3)	0 (0%)
B	51 (36,4)	24 (52,2)	27 (28,7)
C	87 (62,1)	20 (43,5)	67 (71,3)
*MELD score mean (SD)*	21,24 (9,1)	18,9 (13)	22,80 (9,16)
*Serum tests median (IQR)*			
Albumin (g/dL)	2,53 (0,54)	2,60 (0,8)	2,40 (0,7)
Total bilirubin (mg/dL)	2,60 (7,14)	2,40 (3,5)	3,00 (5,4)
INR	1,91 (1,14)	1,49 (0,43)	1,67 (0,75)
Creatinine (mg/dL)	1,41 (1,53)	1,27 (1,38)	1,55 (1,5)
*Ascites tests mean (SD)*			
Neutrophils count	3970 (8657)	56 (68)	5946 (10071)
Albumin in ascites (g/dL)	0,57 (0,42)	0,54 (0,47)	0,58 (0,4)
Total protein in ascites (g/dL)	1,27 (0,9)	1,10 (0,87)	1,36 (0,91)
Ascites to serum albumin gradient	1,97 (0,59)	2,16 (0,62)	1,89 (0,55)
*Time between admission and paracentesis mean (SD)*			
<48h	77 (52,7)	22 (44,9)	42 (49)
48-72h	7 (4,8)	1 (2)	14 (16)
>72h	62 (42.5)	36 (37,1)	30 (35)
*Hospitalization in previous 30 days mean (SD)*	49 (33,8)	17 (34,7)	32 (33,3)
*Antibiotic prophylaxis agains SBP mean (SD)*	28 (19)	9 (18)	19 (20)

SBP: spontaneous bacterial peritonitis; SD: standard deviation; IQR: interquartile range; MDR: multidrug resistant; XDR: extensively drug resistant.

**Table 2:** Bacterial resistances of most prevalent germs against specific antibiotics among all cultures.

Microorganism	Acinetobacter (n=6)	E. coli (n=49)	Enterobacter (n=7)	Enterococcus (n=23)	Klebsiella pneumoniae (n=27)	S. aureus (n=31)	Pseudomonas sp. (n=4)	Coag-negative Staphylococcus (n=19)	All germs (n = 183)
Resistance category (%)									
MDR	33,3%	53,1%	28,6%	17,4%	18,5%	38,7%	---	47,4%	32,2%
XDR	50,0%	12,2%	14,3%	0,0%	22,2%	9,7%	---	0,0%	10,3%
Specific resistances (%)							---		
Oxacillin	---	---	---	---	---	45,2%	0,0%	42,1%	12,0%
Vancomycin	---	---	---	17,4%	---	---	0,0%	5,3%	2,7%
Clindamycin	---	---	---	---	---	64,5%	---	42,1%	15,8%
Gentamycin	66,7%	14,3%	0,0%	4,3%	25,9%	41,9%	---	36,8%	21,9%
Amicacin	100,0%	2,0%	0,0%	---	3,7%	---	25,0%	-	5,5%
Ampicillin	---	69,4%	---	34,8%	---	---	---	-	24,0%
Ampicillin-Sulbactam	66,7%	34,7%	57,1%	---	44,4%	---	0,0%	-	26,8%
Piperacillin-Tazobactam	100,0%	4,1%	14,3%	---	33,3%	---	25,0%	-	13,1%
Cefuroxime	---	24,5%	42,9%	---	40,7%	---	0,0%	-	23,5%
Cefotaxime	---	10,2%	0,0%	---	25,9%	---	25,0%	-	8,7%
Ceftazidime	100,0%	12,2%	0,0%	---	37,0%	---	0,0%	-	14,8%
Ceftriaxone	100,0%	22,4%	28,6%	100,0%	37,0%	45,2%	0,0%	0,0%	39,3%
4th gen.cephalosporin	100,0%	14,3%	0,0%	---	40,7%	---	0,0%	-	14,8%
Levofloxacin/Ciprofloxacin	100,0%	36,7%	14,3%	17,4%	25,9%	35,5%	---	26,3%	30,1%
Imipenem	---	2,0%	0,0%	0,0%	7,4%	---	---	-	1,6%
Meropenem	100,0%	0,0%	0,0%	---	14,8%	---	---	-	5,5%
Trimethoprim-Sulfamethoxazole	83,3%	61,2%	14,3%	---	37,0%	12,9%	---	26,3%	31,7%

**Table 3:** Bacterial resistances of most prevalent germs against specific antibiotics among SBP-related cultures.

Microorganism	Acinetobacter (n=2)	E. coli (n=38)	Enterobacter (n=4)	Enterococcus (n=10)	Klebsiella pneumoniae (n=18)	S. aureus (n=12)	Serratia sp. (n=2)	Coag-negative Staphylococcus (n=19)	All germs (n=113)
Resistance category									
MDR	0,0%	60,5%	25,0%	20,0%	22,2%	41,7%	0,0%	47,4%	39,8%
XDR	100,0%	2,6%	25,0%	0,0%	16,7%	0,0%	0,0%	0,0%	7,1%
Specific resistances									
Oxacillin	-	-	-	-	-	41,7%	-	42,1%	11,5%
Vancomycin	-	-	-	20,0%	-	-	-	5,3%	2,7%
Clindamycin	-	-	-	-	-	58,3%	-	42,1%	13,3%
Gentamycin	50,0%	5,3%	0,0%	0,0%	16,7%	41,7%	0,0%	36,8%	17,7%
Amicacin	100,0%	2,6%	0,0%	-	5,6%	-	0,0%	-	4,4%
Ampicillin	-	68,4%	-	20,0%	-	-	-	-	26,5%
Ampicillin-Sulbactam	100,0%	28,9%	50,0%	-	44,4%	-	100,0%	-	30,1%
Piperacillin-Tazobactam	100,0%	2,6%	25,0%	-	27,8%	-	0,0%	-	13,3%
Cefuroxime	0,0%	21,1%	50,0%	-	33,3%	-	-	-	29,2%
Cefotaxime	0,0%	5,3%	0,0%	-	22,2%	-	-	-	7,1%
Ceftazidime	100,0%	7,9%	0,0%	-	33,3%	-	0,0%	-	11,5%
Ceftriaxone	100,0%	21,1%	50,0%	100,0%	27,8%	41,7%	-	0,0%	39,8%
4th gen.cephalosporin	100,0%	13,2%	0,0%	-	33,3%	-	0,0%	-	13,3%
Levofloxacin/Ciprofloxacin	100,0%	28,9%	0,0%	20,0%	16,7%	33,3%	0,0%	26,3%	25,7%
Imipenem	0,0%	2,6%	0,0%	0,0%	5,6%	-	-	-	1,8%
Meropenem	100,0%	0,0%	0,0%	-	11,1%	-	0,0%	-	4,4%
Trimethoprim-Sulfamethoxazole	100,0%	57,9%	0,0%	-	22,2%	8,3%	0,0%	26,3%	32,7%

SD: standard deviation; IQR: interquartile range

^*∗*^Specific data from another 8 cultures are not shown in this table and consisted of multisensitive Klebsiella oxytoca, Proteus mirabilis, Pseudomonas aeruginosa, Salmonella sp., S. pneumoniae, and S. viridans.

## Data Availability

The whole data used to support the findings of this study are restricted by the Hospital de Clinicas de Porto Alegre Ethics Board in order to protect patient privacy. Data are available from Dr. Jerônimo De Conto Oliveira, jeronimodco@gmail.com, for researchers who meet the criteria for access to confidential data.
